# Brain Monitoring in Critically Neurologically Impaired Patients

**DOI:** 10.3390/ijms18010043

**Published:** 2016-12-27

**Authors:** Salazar Jones, Gary Schwartzbauer, Xiaofeng Jia

**Affiliations:** 1 Department of Neurosurgery, University of Maryland School of Medicine, Baltimore, MD 21201, USA; SAJones@som.umaryland.edu (S.J.); GSchwartzbauer@som.umaryland.edu (G.S.); 2 R Adams Cowley Shock Trauma Center, University of Maryland School of Medicine, Baltimore, MD 21201, USA; 3 Institute for Global Health, University of Maryland School of Medicine, Baltimore, MD 21201, USA; 4 Department of Orthopaedics, University of Maryland School of Medicine, Baltimore, MD 21201, USA; 5 Department of Anatomy and Neurobiology, University of Maryland School of Medicine, Baltimore, MD 21201, USA; 6 Department of Biomedical Engineering, The Johns Hopkins University School of Medicine, Baltimore, MD 21205, USA; 7 Department of Anesthesiology and Critical Care Medicine, The Johns Hopkins University School of Medicine, Baltimore, MD 21205, USA

**Keywords:** brain monitoring, cerebral microdialysis, serum biomarker, pressure reactivity index, brain tissue oxygenation, optogenetics, multimodality monitoring

## Abstract

Assessment of neurologic injury and the evolution of severe neurologic injury is limited in comatose or critically ill patients that lack a reliable neurologic examination. For common yet severe pathologies such as the comatose state after cardiac arrest, aneurysmal subarachnoid hemorrhage (aSAH), and severe traumatic brain injury (TBI), critical medical decisions are made on the basis of the neurologic injury. Decisions regarding active intensive care management, need for neurosurgical intervention, and withdrawal of care, depend on a reliable, high-quality assessment of the true state of neurologic injury, and have traditionally relied on limited assessments such as intracranial pressure monitoring and electroencephalogram. However, even within TBI there exists a spectrum of disease that is likely not captured by such limited monitoring and thus a more directed effort towards obtaining a more robust biophysical signature of the individual patient must be undertaken. In this review, multimodal monitoring including the most promising serum markers of neuronal injury, cerebral microdialysis, brain tissue oxygenation, and pressure reactivity index to access brain microenvironment will be discussed with their utility among specific pathologies that may help determine a more complete picture of the neurologic injury state for active intensive care management and long-term outcomes. Goal-directed therapy guided by a multi-modality approach appears to be superior to standard intracranial pressure (ICP) guided therapy and should be explored further across multiple pathologies. Future directions including the application of optogenetics to evaluate brain injury and recovery and even as an adjunct monitoring modality will also be discussed.

## 1. Introduction

Brain monitoring is important in critical clinical scenarios where the extent or evolution of neurologic injury is unknown. Common situations include the comatose state after cardiac arrest, poor grade aneurysmal subarachnoid hemorrhage (aSAH), and severe traumatic brain injury (TBI) et al. The extent of neurologic injury and injury progression are crucial in determining prognosis and guiding intensive care strategies to ameliorate the neurologic injury [1,2].

By examining several clinical diagnoses, a glimpse of the scale of critically ill patients in whom brain monitoring is of importance can be seen. Each year, around 235,000 patients are hospitalized in the United States with traumatic brain injury with a significant portion having severe TBI (Glasgow Coma Scale score ≤ 8) [3]. Around 300,000 individuals have out-of-hospital cardiac arrests each year [1]. Of those comatose after cardiac arrest, half will not regain consciousness [4]. In the United States, about 30,000 persons each year have an aneurysmal subarachnoid hemorrhage with mortality close to 50% [5]. The impact of brain monitoring lies in the common thread among these pathologies. There is an initial inciting event that causes primary neurologic injury of unknown degree, followed by dynamic pathophysiological changes causing secondary neurologic injury. In the comatose or sedated patient, even the most attentive clinicians can often be in the dark about evolving secondary neurologic injury.

## 2. Objective of Brain Monitoring

Early determination of neurologic prognosis would decrease situations of prolonged medical resource utilization that does not provide any additional clinical benefit to the patient, but more importantly avoid withdraw of life support prematurely for those patients that may have a chance to recover later. As a supplement to the neurologic examination, other testing including electrophysiology markers such as electroencephalography (EEG) [6,7,8,9,10] and somatosensory evoked potentials [11,12] developed from preclinical studies with or without quantitative methods have been used in determining clinical prognosis after cardiac arrest patients treated with targeted temperature management [1,2]. In patients undergoing therapeutic hypothermia, sedation and neuromuscular blockade limit the ability of clinicians to obtain a reliable neurologic exam. For such patients, the main mechanisms of injury—ischemia and subsequent reperfusion injury to the brain—occur at the time of cardiac arrest and return of circulation [13]. As such, it is reasonable to seek methods to determine prognosis earlier in the care of comatose post-cardiac arrest patients. The information obtained would allow for more appropriate communication with patients’ families to aid in goals of care.

In other pathologies such as aSAH and TBI, there is both an initial primary injury and secondary injury that occurs over the ensuing weeks. The evolving secondary injury may often predominate as the most significant determinant of neurologic outcome [14]. This is especially the case when such evolution requires interventions such as decompressive craniectomy in severe TBI patients or endovascular therapy for vasospasm in aSAH patients. Critical patients in the intensive care unit (ICU) also are subject to additional systemic insults causing secondary neurologic injury including fever, respiratory failure, anemia, seizure, and infection [15]. Each insult can place patients on a lower recovery trajectory, hence, measures are taken to address any systemic insult. However, a more refined approach is needed to identify and address changes in the local brain environment that occur without a systemic correlate. Multiple techniques for brain monitoring are available (See Table 1).

In this review, we focus on techniques such as serum markers of neuronal injury, intracranial pressure monitoring, microdialysis, pressure reactivity index, and brain tissue oxygenation that are currently available at the bedside for clinical decision making. Optogenetics is also presented as the potential future of brain monitoring. Though the following techniques are discussed individually, the astute clinician will utilize multiple methods to generate a more complete clinical picture. Available multimodality monitors are capable of probes for microdialysis, brain tissue oxygenation, and intracranial pressure monitoring.

## 3. Serum Markers of Glial or Neuronal Injury

In the area of severe neurologic injury, serum markers of glial or neuronal injury are being investigated as measures of neurologic injury and prognosis.

Neuron specific enolase (NSE) is a glycolytic enzyme expressed in the cytosol of neuronal and glial cells. It is unique in that it has additional functions in the inflammatory response to injury after spinal cord injury, hypoxic-ischemic brain injury, and traumatic brain injury [17]. NSE goes to the cell surface and serves as a receptor to the serine protease plasminogen to stimulate degradation of the extracellular matrix [17]. Rech et al. [18] demonstrated in 45 post-cardiac arrest patients, that higher NSE levels within 48 h of arrest were associated with unfavorable outcomes at 6 months and suggested a cut-off of 60 ng/mL to have 100% specificity, albeit 35% sensitivity, in predicting unfavorable outcomes. In out-of-hospital cardiac arrest patients, Stammet et al. [19] demonstrated in 686 patients randomized to targeted therapeutic hypothermia at 33 or 36 °C that serial NSE levels were not affected by hypothermia and were higher at all measurements within 72 h in patients with unfavorable outcomes at 6 months. A cut-off of 45 ng/mL at the 72 h time point had a specificity of 99% and sensitivity of 54% to predict an unfavorable outcome. In aSAH, Tawk et al. [20] found patients with NSE levels >15 ng/mL had a moderate correlation with worse clinical severity and greater disability at discharge. In TBI, NSE has a less clear role. Higher levels of NSE are associated with unfavorable outcomes, but a specific laboratory cutoff value has not been determined and NSE has poor discrimination among the unfavorable outcomes [21]. Limitations of NSE testing are extracranial sources of NSE [22] and surgery can increase the levels of serum NSE [23].

Another serum marker of interest is S100B. S100B is a calcium-binding protein present in cytosol of astrocytes important for astrocyte activation in brain injury repair [24]. In the area of cardiac arrest, elevated S100B levels in patients correlate with severity of neurologic injury with persistently elevated levels >0.414 ng/mL at 72 h predicting mortality with 100% specificity [25,26]. In aSAH, elevated S100B levels aren’t able to predict delayed cerebral ischemia that can occur from vasospasm but are associated with worse Glasgow Outcome Score at 6 months [27,28]. In severe TBI, S100B levels 12 h from the time of trauma are predictive of Glasgow Outcome Score at 1 year [29]. S100B levels are subject to extracranial sources as well which presents limitations in multi-trauma patients and post cardiac arrest patients [22,30]. Of note for those with severe TBI, surgery or insertion of an external ventricular drain can affect serum levels of S100B [23,31].

Glial fibrillary acidic protein (GFAP), an astrocytic intermediate filament, has also been investigated serum marker of neuronal injury. In the area of cardiac arrest, GFAP levels are generally elevated in patients with poor outcomes, but there is inadequate discrimination among unfavorable outcomes to truly aid in predicting specific outcomes [32,33]. In aSAH patients, elevated levels of GFAP on admission correlate with severity of aSAH and represented a ten times more likelihood of poor outcome at the time of hospital discharge [34]. GFAP levels were elevated with secondary events such as aneurysm re-bleed and moderately correlated with poor outcome at 1 year (*r* = −0.48) [35]. Using a serum cutoff value of 0.15 µg/L to classify elevated GFAP levels was determined to be highly sensitive (92%) but not very specific (40%) in predicting unfavorable outcomes in aSAH [35]. In severe TBI, GFAP levels on admission moderately correlate with severity on trauma but is not as clinically useful in predicting outcome [36]. However, an elevated GFAP level (>11.14 ng/mL) at day 7 is an independent predictor of unfavorable outcome at 1 year (sensitivity 81.8%, specificity 88.9%) [37]. In patients with mild TBI, an elevated GFAP can predict those patients that are likely to have intracranial lesions on computed tomography (receiver-operator characteristics AUC 0.84) [38]. Papa et al. [39] showed that GFAP levels could predict which patients with mild to moderate TBI would need a neurosurgical intervention within the 7 days after trauma.

## 4. Cerebral Blood Flow Monitoring

Abnormalities in cerebral blood flow (CBF) are central to pathologies such as vasospasm in aSAH, traumatic brain injury, and in cardiac arrest. Direct clinical use in the intensive care unit are in optimizing a patient’s blood pressure, diagnosing vasospasm in aSAH, and for brain death examination. There are several methods for monitoring cerebral blood flow including: transcranial doppler, perfusion computed tomography (CT), diffusion correlation spectroscopy, and laser speckle imaging. Transcranial doppler is a well understood modality that assesses blood velocity in the major intracranial vessels and can be performed at the bedside. Intuitively, absence of velocity is seen in the absence of flow, however, relying on a negative finding can be problematic given operator dependence. In addition, only the major intracranial vessels are amenable to doppler investigation which may not be representative of the microvascular circulation [40]. The information obtained constitutes a single data point in time as opposed to a continuous monitoring tool. Moreover, in vasospasm for aSAH, the correct interpretation is to suspect decreased flow in the presence of elevated velocities. When compared to cerebral blood flow measured using continuous arterial spin labeling magnetic resonance imaging (MRI) in healthy patients, transcranial doppler velocities had no correlation [40].

Perfusion CT is a well-accepted and widely used method for measuring cerebral blood flow. Obtaining a perfusion CT requires the transport of critically injured patients to a CT scanner elsewhere in the hospital. The data obtained, although useful, still constitutes one data point in time, thus not suitable as a monitor. Near-infrared spectroscopy (NIS) takes advantage of differences in spectra of hemoglobin bound (HbO_2_) and unbound to oxygen to measure changes in HbO_2_ concentration, thus a surrogate of cerebral blood flow. NIS is a non-invasive method involving optical probes placed on the scalp with a maximum depth of tissue of approximately 2–3 cm [41]. NIS has been used as a research tool to study cerebral blood flow and cerebral metabolism in TBI [42]. As a measure of cerebral oxygenation, NIS is likely still inferior to invasive PbtO_2_ monitoring [43]. In the aSAH population, NIS has been used to monitor efficacy of vasospasm treatment [44]. Diffuse correlation spectroscopy (DCS) represents a non-invasive method of using optical probes to continuously measure changes in blood flow, thus measuring a relative CBF. DCS has been shown in pre-clinical models to identify cerebral blood flow changes in line with perfusion CT [45]. DCS combined with NIS has been validated in the neuro-intensive care unit with a good correlation of cerebral blood flow findings compared to xenon enhanced perfusion CT (*r* = 0.73) [46]. More clinical studies will be needed to determine if DCS or combined DCS/NIS can predict or identify early patients with vasospasm in aSAH.

Laser speckle imaging (LSI) is another non-invasive technique to image cerebral vasculature. LSI relies on the scattering of photons when photons strike particles in motion (e.g., red blood cells) [47,48]. LSI has been used in the pre-clinical research models to image the vasculature in response to hypothermia, peripheral electrical stimulation and for real-time monitoring of cerebral blood flow in rats after cardiac arrest [49,50,51]. LSI is still an emerging technology for clinical use, while translational researches are moving this technology from bench to bedside [52]. LSI is limited by the lack of depth resolution. Currently, LSI can be used after a window in the skull has been created. Intraoperative LSI during neurosurgical procedures have properly shown increase blood flow after bypass procedures and decrease in blood flow after coagulation of cortical vessels [53,54]. Further development is needed to advance incorporation of LSI when an intact human skull is in place to supplant LSI as a monitoring tool.

## 5. Intracranial Pressure Monitoring

Intracranial pressure (ICP) monitoring is a well-recognized method of monitoring for intracranial changes that can portend neurological worsening. The development of mass lesions including intracranial hemorrhage and cerebral edema are accompanied by a rise in intracranial pressure. If needed in severe TBI, decompressive craniectomy in cases of persistently elevated ICPs can be performed to increase overall survival [55]. As such, ICP monitoring has a well-defined role in TBI as recommended by the Brain Trauma Foundation [56]. Placement of an ICP monitor requires an invasive procedure for placement of a probe most commonly within the parenchyma or catheter within the ventricles. The use of an intraventricular catheter is advantageous for its ability to remove CSF as a therapy. It is not firmly established that the institution of ICP monitoring for severe TBI patients improves long-term outcomes [57]. Results from the Benchmark Evidence from South American Trials: Treatment of Intracranial Pressure (BEST TRIP) trial suggest that ICP monitoring in TBI is no better than clinical examination and CT imaging for outcomes 14-day and 6-month mortality [58]. Other reports have suggested the use of ICP monitoring helps to identify those TBI patients that need craniotomy/craniectomy and reduces in-hospital mortality [57,59]. In aSAH, elevated ICP is associated with worse outcomes, but strong data showing the prognostic value of ICP monitoring is lacking [60].

ICP monitoring has several limitations. Not all pathologies have the potential for the development of a mass lesion and, thus, a rise in ICP. The comatose state after cardiac arrest is an example where ICP monitoring does not have a clear role [61]. In addition, a rise in ICP is non-specific and may be secondary to dysfunctional autoregulation or hyperemia. Clinical suspicion of worrisome pathology based on a rise in ICP has to be confirmed by imaging. ICP monitoring, however, can be combined with other forms of cerebral monitoring for a more complete neurologic picture.

## 6. Cerebral Microdialysis—Assessment of Brain Microenvironment

Cerebral microdialysis is a form of invasive monitoring that requires insertion of a small catheter into the brain parenchyma. Invasive monitors often are inserted into the right frontal lobe. Endogenous molecules such as glucose, neurotransmitters, and products of metabolism are allowed to equilibrate within the dialysate. The concentration of endogenous molecules can then be measured from the dialysate. Common microdialysis analysis measures glutamate, glucose, lactate, and pyruvate.

Glutamate is an excitatory neurotransmitter that can increase brain activity and metabolic requirements. Increased cerebral glutamate has been seen in cardiac arrest and aSAH and implicated in ischemia and mediating apoptosis of neurons [62,63,64]. The excitatory neurotransmitter glutamate in high amounts can lead to excitotoxicity via spreading depolarizations that lead to increased energy substrate depletion and anaerobic metabolism. Hinzman et al. [65] demonstrated by placing a subdural electrode strip adjacent to a microdialysis catheter in 16 severe TBI patients that increasing glutamate was seen with increasing frequency of spreading depolarizations and metabolic crisis.

Lactate and pyruvate are two byproducts of metabolism. Pyruvate is the end product of glycoysis. In the setting of anaerobic respiration, pyruvate is converted to lactate. The ratio of lactate to pyruvate gives information about the overall state of oxygen delivery for cellular aerobic respiration. An elevated lactate to pyruvate ratio (>40) is worrisome for a metabolic crisis with possible causes including ischemia, hypoxia, low glucose availability and mitochondrial dysfunction. Elevated lactate to pyruvate ratios indicating ischemia have been observed after cardiac arrest [62]. Of note, lactate by itself may be elevated due to excessive glycolysis. Oddo et al. [66] showed that a state of cerebral hyperglycolysis was shown to be predictive of a good 6-month outcome in aSAH patients.

A decrease in cerebral blood flow can lead to ischemia and subsequently a metabolic crisis. Rostami et al. [67] also showed in aSAH patients that high lactate to pyruvate ratios were associated with decreased cerebral blood flow around the microdialysis catheter as seen on Xenon enhanced computed tomography.

**Figure 1:**
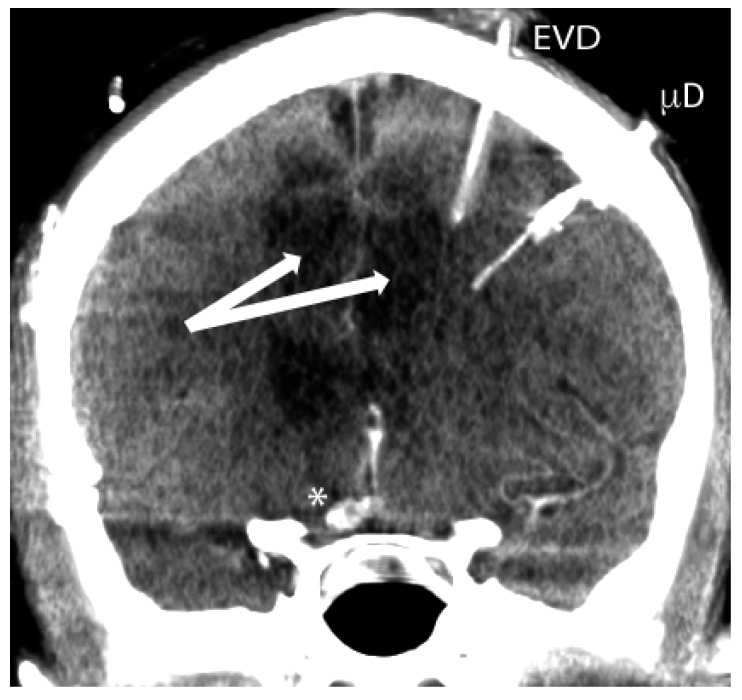
Computed tomography showing External ventricular drain (EVD) and microdialysis (µD) probe in place in aSAH patient. Sometimes the multimodality monitoring probes are in proximity to injured brain, but often they are not. Severe vasospasm resulting in ischemic brain (arrows) occurred days after anterior communicating artery aneurysm clipping (*).

An argument against the utility of microdialysis stems from the dialysate being taken from a single area of the brain. The brain microenvironment in one region may not be reflective of the global cerebral environment. This can be especially true in aSAH patients where vasospasm may be limited to individual vascular territories distant from the microdialysis monitor (See Figure 1). However, this assertion against microdialysis has been challenged in pathologies that involve diffuse injury such as in TBI. Bouzat et al. [68] used examined computed tomography perfusion studies of 27 severe traumatic brain injury patients with microdialysis monitoring. Regional blood flow around the catheter correlated with global cerebral blood flow [68]. In addition, low dialysate glucose and elevated lactate to pyruvate ratio >40 significantly predicted low regional cerebral blood flow (<35 mL/100 g brain tissue/min) on CT perfusion [68]. Moreover, the addition of cerebral microdialysis and PbtO_2_ monitoring to standard ICP monitoring was significantly more accurate in predicting low regional cerebral blood flow than ICP monitoring alone [68].

Using cerebral microenvironment metabolic profiles, the data has shown to be useful in predicting neurologic prognosis. In severe TBI patients, Timofeev et al. [69] showed that microdialysis markers were independently associated with outcome. In his prospective cohort of 223 patients, elevated levels of glutamate (>10 µmol/L) and high lactate to pyruvate ratios (>25) were associated with higher mortality. Conversely, favorable outcomes were more likely in patients with lower lactate/pyruvate ratios and total lactate levels [69]. Pyruvate was an independent negative predictor of mortality [69].

In addition to low glucose availability that can precipitate a metabolic crisis, fluctuations in glucose appear to be detrimental. Kurtz et al. [70] showed that patients with poor grade aSAH and high systemic glucose variability were more likely to show signs of metabolic distress defined as lactate to pyruvate ratio >40 on cerebral microdialysis analysis and had higher in-hospital mortality. Similarly, Gupta et al. [71] demonstrated in severe TBI patients that required a decompressive craniectomy, patients with more episodes of systemic hypoglycemia and hyperglycemia during the acute phase had a poor Glasgow Outcome Scale score at 3 months. The poor outcome group also had more episodes of cerebral hypoglycemia, although a temporal correlation of blood and cerebral glucose levels was not seen [71]. Rostami and Bellander showed with cerebral microdialysis that blood glucose levels and cerebral glucose levels may correlate in non-injured brain but not in injured brain [72]. To our knowledge, the role of cerebral glycemic variability using cerebral microdialysis in post cardiac arrest comatose patients is not known. Cerebral glucose metabolism as assessed by positron emission tomography (PET), though usually with limited image resolution and only when the patient’s condition is stable and allows for the exam, has shown decreased radiolabeled glucose uptake in patients in persistent vegetative state after cardiac arrest, although the utility in predicting prognosis is indeterminate [73,74]. However, in general, systemic glycemic variability is known to be a predictor of mortality in patients in the intensive care unit [75]. In addition, decreased brain to serum glucose ratios correlate with in-hospital mortality and poorer outcomes across multiple pathologies including aSAH, cardiac arrest, intracerebral hemorrhage, and TBI [76].

Additional markers have been investigated using microdialysis. Brain extracellular potassium concentration ([K^+^]) is a marker for metabolic distress on microdialysis and poor 3-month outcome in poor grade aSAH patients [77]. The brain extracellular [K^+^] does not necessarily correlate with serum [K^+^] signifying such useful information is only available through the use of microdialysis [77].

Newer techniques have allowed for the continuous real-time assessment of highly reactive oxygen species known to lead to cellular injury and necrosis in pre-clinical models [78]. Elevated cerebral dialysate levels of interleukin-6 on admission were associated with delayed cerebral ischemia in poor grade aneurysmal subarachnoid hemorrhage patients [79]. Levels of matrix-metalloproteinase-9 were significantly elevated in patients presenting with poor grade aSAH [79]. These markers of a pro-inflammatory state are uniquely available to microdialysis techniques and independent of traditional systemic markers of inflammation including white blood cell count, C-reactive protein, and body temperature [79]. Further work will allow for the quantification and eventual therapy for injurious oxidative stress.

Logistically, the use of microdialysis can be labor intensive. In our neurocritical care unit, the dialysate is analyzed hourly and requires a critical care nurse dedicated only to the single patient with a microdialysis catheter. In addition, the invasive nature introduces inherent risks including hemorrhage, infection, and hardware malfunction. Nevertheless, microdialysis offers a window into the individual’s pathophysiology underlying their cerebral injury and insults leading to secondary injury. The full transformative effect of microdialysis in the neurocritical care has yet to be seen.

## 7. Pressure Reactivity Index

Pressure reactivity index (PRx) is a measure of the correlation of blood pressure and intracranial pressure. In the normal range of intact autoregulation, blood pressure and ICP are negatively correlated. That is, an increase in cerebral blood flow is followed by cerebral vasoconstriction that decreases the volume of the intracranial blood compartment, thereby decreasing the ICP.

PRx requires an ICP monitor to correlate the ICP with the blood pressure. For severe TBI patients (GCS ≤ 8) with an abnormal CT scan, ICP monitoring is recommended by the Brain Trauma Foundation guidelines [56]. As such, for the severe TBI population, PRx provides critical autoregulation information without the need for additional invasive devices.

With PRx, clinicians can identify the individualized blood pressure range in which autoregulation is intact whereby cerebral perfusion pressure (CPP) is preserved. The individual patient’s optimal CPP (CPPopt) is the CPP at which the PRx is most negative, that is, when autoregulation is most appropriately responsive. Aries et al. [80] showed in a large retrospective cohort of severe TBI patients requiring neurocritical care that six-month Glasgow Outcome Scores were better in patients whose CPP was maintained within 5 mmHg of their CPPopt. Mortality was greatly increased in patients whose CPP was more than 5 mmHg below their CPPopt [80]. In a follow-up single center prospective pilot study, Dias et al. [81] demonstrated the feasibility of CPPopt guided care, and reconfirmed that patients with a median CPP 6.6 mmHg below CPPopt had poorer Glasgow Outcome Scores at 6 months. PRx has been investigated in the aSAH population. Despite case reports of PRx being useful for predicting delayed cerebral ischemia, its utility is not firmly established [82]. PRx may be able to identify patients with decreased cerebral blood flow, but it has not been shown to reliably predict delayed cerebral ischemia [83]. The Neurocritical Care Society guidelines acknowledge the multiple methods for monitoring cerebral autoregulation, but currently give a “weak recommendation” to their use [84]. Since no technique is clearly better than others, PRx is advantageous in patients already with ICP monitoring since no additional invasive procedure or blood draw is needed. To our knowledge, PRx has not been evaluated in the adult post-cardiac arrest patient. Limitations of PRx include the need for additional software that can compute CPPopt. In addition, the software may not be able to determine CPPopt for all patients [81].

## 8. PbtO_2_ Monitoring

PbtO_2_ monitoring is an invasive form of monitoring the brain tissue oxygenation via a catheter inserted into brain parenchyma. As for any form of invasive monitor, risks of hemorrhage, infection, and device malfunction are possible.

The level of brain tissue oxygenation is a crucial measure in the intensive care unit. A PbtO_2_ < 15 mmHg is associated with cerebral ischemia and poor neurological outcome. Normal PbtO_2_ is above 20 mmHg. ICU maneuvers to increase PbtO_2_ include pressors or volume expanders to increase mean arterial pressure (thereby, cerebral perfusion pressure), ICP reduction, blood transfusion to increase oxygen carrying capacity, and by increasing the fraction of inspired oxygen. Increasing the oxygen carrying capacity by blood transfusion increases the PbtO_2_ in as little as 2 h [85]. In poor grade aSAH patients that underwent intra-arterial spasmolysis, a rise in PbtO_2_ after treatment correlated with the angiographic response of improved blood flow [86]. The availability of measures to affect brain tissue oxygenation reinforces the clinical utility of real-time PbtO_2_ monitoring.

Spiotta et al. [87] noted that in a cohort of 70 patients with severe TBI that had goal directed PbtO_2_ therapy (PbtO_2_ > 20 mmHg) in addition to standard ICP/CPP management had significantly lower 3-month mortality—25.7% compared to 45.3% in standard ICP/CPP therapy group. At 3 months, the PbtO_2_ goal directed therapy group had significantly more favorable outcome rates (64.3% compared to 39.6%) [87]. Other groups have similarly showed a decrease in mortality and improved 6-month GOS outcomes with the addition of goal directed PbtO_2_ therapy over standard ICP/CPP therapy alone [88,89]. In aSAH, PbtO_2_ monitoring has less of a role in predicting vasospasm or outcome [90]. The utility of PbtO_2_ in comatose state after cardiac arrest is not known and currently not recommended [61].

Limitations of PbtO_2_ monitoring are inherent to the regional monitoring. The utility will depend on the pathology. Severe TBI as a global process lends itself as a clinical entity where PbtO_2_ monitoring would be of value.

## 9. Timing of Brain Monitoring

With the multitude of methods available for brain monitoring, timing of each has to be considered. In our view, the timing depends on the primary diagnosis. For the post-cardiac arrest comatose state, the serum markers of neuronal injury NSE and S100B within 48–72 h are useful for prognostication [18,19,25]. As the neurologic injury is predominantly subsequent to post cardiac arrest hypoxic state, invasive methods to detect secondary injury are less warranted. Electrophysiological testing and magnetic resonance imaging are additional methods for assessing prognosis 72 h after cardiac arrest [1].

In poor grade aSAH patients, the utility of serum markers of neuronal injury is best within the 24–72 h for predicting unfavorable outcomes [20,28,34]. S100B peaks early within 24 h and then decreases [27]. GFAP was shown to peak around day 2 [35]. For aSAH patients, preventing and treating delayed ischemic neurologic deficits from cerebral vasospasm is a primary objective in the intensive care setting. Delayed ischemia is very difficult to detect on the neurologic examination in the poor grade aSAH patient. Given the peak vasospasm window occurs between days 4 to 11 after aneurysm rupture [91], the use of microdialysis to detect regional ischemia may be targeted to this time period. Additional methods specifically for detecting vasospasm must be employed given not all cerebral vascular territories are affected equally.

In severe traumatic brain injury, S100B levels within 24 h of injury and an elevated GFAP level at day 7 are useful for outcome prediction [27,29,37]. ICP monitoring should be followed on admission as recommended by the Brain Trauma Foundation [56]. Beyond prognostic purposes, the use of other modalities such as microdialysis, PbtO_2_, and pressure reactivity index may improve outcomes (See Table 2) and can be considered where available.

## 10. Optogenetics—Possible Future Monitoring Tool?

Optogenetics is an interesting area of research relatively in its infancy but may have potential clinical applications in brain monitoring. In its simplest permutation, optogenetics involves optically recording changes in membrane potential. Regarding the central nervous system, changes in membrane potential is how action potentials spread down an axon, across a synapse, and activate second order neurons. The ability to see neuronal signal transmission in action implicates that functional circuitry can be characterized. The functional assessment possible through optogenetics is an advantage over current brain monitoring techniques.

The vehicle for gaining such information lies in the utilization of genetically encoded voltage indicators that fluoresce with changes in membrane potential. Since their introduction, voltage indicators have become much more sophisticated with voltage range specificities that allow for differentiating between axon depolarization, sub-threshold membrane potential changes, and synaptic activity as recently reviewed [92]. Optical recording of neural activity has been performed in in vitro cell models, whole brain explant models, and in vivo pre-clinical models [93,94]. Optogenetics can be combined with other therapies to monitor for improvement in neuronal functioning. Jayaprakash et al. [95] demonstrated the ability to optically monitor for synapse formation in an experimentally treated preclinical model of spinal cord injury. A current limitation of optogenetics is that the brain tissue of interest has to be genetically targeted for optogenetic monitoring. For the adult human brain, optogenetic monitoring is likely to be limited to specific regions or functional pathways rather than the entire brain.

Though seemingly a leap at this time, optogenetics may allow for in-depth real time brain monitoring of functional circuitry. Considerable work remains in the areas of voltage indicator optimization, integration and targeted gene expression, optical recording, and scaling to the human brain. Since optogenetic monitoring will be limited to the area of interest, it will complement current methods of brain monitoring to provide a better overall neurologic injury picture for monitoring, treatment, and prognosis.

## 11. Use of Multimodality Monitoring

There are multiple modalities of brain monitoring complement each other to provide a better picture of neurologic injury. With a better understanding of a patient’s individual cerebral pathophysiology, multimodality approach can lead to goal directed therapy to optimize the individual patient’s physiology. The use of goal-directed therapy guided by brain monitoring tools requires further clinical evaluation to identify improvements in outcomes. For severe TBI, several modalities have been combined with standard ICP monitoring with improvements in outcomes (See Table 2). Larger clinical series are needed to lead to widespread adoption of multimodality monitoring.

## 12. Conclusions

Tools for brain monitoring are becoming more sophisticated and continually improving to allow for real-time individualized brain monitoring. The role of microdialysis will only increase as markers of initial and ongoing neuronal injury are researched and applied to the clinical arena. It is conceivable that different pathologies may require different microdialysis markers on injury, thus further individualizing patient care. The availability and further development of multimodality monitors allow for concurrent brain tissue oxygenation, intracranial pressure monitoring, and cerebral microdialysis. The role of optogenetics in evaluating brain injury and recovery towards possible brain monitoring is seemingly far away, but may fundamentally change how we monitor the brain after injury. The importance of neurocritical care management is seen with improved functional outcomes for patients undergoing advanced brain monitoring.

## Figures and Tables

**Table 1:** Techniques for brain monitoring.

Technique	Detail	Advantages	Disadvantages
Neurologic examination	Global assessment of consciousness, sensory and motor responses, cranial nerve assessment, and ancillary tests	Can be used in all patients; findings can direct additional confirmatory testing or imaging	Affected by sedation, fever, seizure; in severely injured, exam may be static despite dynamic neurologic injury
Intracranial pressure (ICP) monitor	Available as ventricular or parenchymal probe	Well-established in traumatic brain injury; Ventricular drain allows removal of cerebrospinal fluid for ICP therapy/labs	Invasive monitor, potential risk of infection, hardware malfunction
Pressure reactivity index (PRx)	Algorithm relates changes in blood pressure to ICP to determine measure of autoregulation	Individualized measure of autoregulation and goal directed cerebral perfusion pressure (CPP) therapy	Requires concurrent ICP monitor; an optimal CPP not always able to be calculated; software malfunction
Microdialysis	Intraparenchymal probe obtains dialysate for lab assays	Informative of cerebral microenvironment and metabolic state; special lab assays possible	Invasive monitor, potential risk of infection, hardware malfunction, results potentially not reflective of entire brain
PbtO_2_	Intraparenchymal probe measures partial pressure of oxygen	Enables targeted therapy	Invasive monitor, potential risk of infection, hardware malfunction, results potentially not reflective of entire brain
Serum markers	Measure of proteins indicative of neuronal injury	Non-invasive prognosticator of outcome	Not “real-time”. Affected by extracranial trauma and timing of injury. Subject to differences in specimen handling, hemolysis and differences between laboratory assays
Continuous electroencephalography	Assess brain electrical activity through scalp electrodes	Cerebral metabolic rate related to brain activity. Despite effects of sedative medications, may still be clinically useful [16]	May be affected by sedation, intracranial mass lesions, presence of other intracranial monitors; inherently subjective interpretation; hardware malfunction; continuous monitoring not widely available
Near-infrared spectroscopy/diffuse correlation spectroscopy	Optical probes placed on scalp. Measures cerebral oxygenation (NIS) and cerebral blood flow (DCS)	Non-invasive, cerebral oxygenation and extraction related to metabolism, cerebral blood flow, continuous monitoring possible	Assess brain tissue to a depth of 2–3 cm, typically frontal lobes

**Table 2:** Use of multimodality monitoring.

Group	Modalities Used	Findings	Comment
Bouzat et al., 2015 [68]	ICP monitor	Multimodality monitoring is more accurate than ICP monitoring alone to predict cerebral hypoperfusion	Multimodality monitoring provides better guidance for the intensivist than ICP monitoring alone
	PbtO_2_		
	cerebral microdialysis		
Spiotta et al., 2010 [87]	ICP monitoring, PbtO_2_	Concurrent ICP/PbtO_2_ targeted therapy resulted in lower mortality (25.7% vs. 45.3%, *p* < 0.05) and improved 3-month favorable outcomes (64.3% vs. 39.6%, *p* = 0.01) compared to ICP monitoring alone	Use of multiple markers of the brain microenvironment improves clinical outcomes
Narotam et al., 2009 [88]		Standard ICP/PbtO_2_ goal directed therapy led to reduction in mortality (25.9% vs. 41.5%) and improved 6-month GOS outcomes over standard ICP/CPP therapy alone	
Lin et al., 2015 [89]		Concurrent goal directed ICP/PbtO_2_ therapy in TBI patients resulted in improved 3 and 6 month mortality compared to ICP therapy alone	
Dias et al., 2015 [81]	ICP monitoring	Patients with unfavorable outcomes at 6 months had a median difference in CPP from CPPopt of −6.6 mmHg, compared with a difference of −1.0 mmHg in the favorable outcomes group (*p* = 0.04)	Individualized goal-directed therapy improves outcomes
	PRx		
